# Cumulative gonadal hormone exposure is nonlinearly associated with risk of canine cranial cruciate ligament disease: a generalised additive model analysis of 20,590 dogs (1988‐2023)

**DOI:** 10.1111/jsap.70023

**Published:** 2025-08-21

**Authors:** D. Low

**Affiliations:** ^1^ Frank. Pet Surgeons IVC Evidensia Leeds UK; ^2^ Swift Referrals IVC Evidensia Wetherby UK

## Abstract

**Objectives:**

To investigate the association between cumulative gonadal hormone exposure and the risk of cranial cruciate ligament disease (CrCLD) in dogs.

**Materials and Methods:**

Secondary analysis of retrospective cohort data of 20,590 dogs (9845 female, 10,745 male) investigating the association between age at gonadectomy and various health outcomes in dogs was conducted. Cumulative gonadal hormone exposure was defined as the continuous independent variable. The occurrence of CrCLD was binarily defined as the outcome measure. Generalised additive models were used to assess the relationship between hormone exposure and risk of CrCLD, adjusted for potential confounders.

**Results:**

The prevalence of CrCLD was 245 of 9845 (2.49%) in females and 203 of 10,745 (1.89%) in males. The risk of CrCLD was nonlinearly associated with cumulative gonadal hormone exposure in both sexes. The risk of CrCLD was greatest in dogs with the least gonadal hormone exposure, sharply decreasing to minima at 1054 days for females and 805 days for males.

**Clinical Significance:**

Risk of CrCLD is nonlinearly associated with age at gonadectomy in dogs. Early gonadectomy may be preliminarily defined as that before 2.9 and 2.2 years in female and male dogs, respectively, in the context of CrCLD risk.

## INTRODUCTION

Cranial cruciate ligament disease (CrCLD) is one of the most common causes of pelvic limb lameness in the dog (Ness et al., 1996; Witsberger et al., 2008), with an estimated prevalence of 0.56% to 2.55% (Adams et al., 2011; Taylor‐Brown et al., 2015; Witsberger et al., 2008). This condition has a complex multifactorial aetiology, with both environmental and genetic risk factors (Comerford et al., 2011) predisposing to degeneration of the cranial cruciate ligament.

Gonad status is an important environmental risk factor that has been previously reported to be associated with CrCLD risk, with gonadectomised dogs overrepresented in most epidemiological studies (Adams et al., 2011; Belanger et al., 2017; Duval et al., 1999; Pegram et al., 2023; Sellon & Marcellin‐Little, 2022). Regarding the timing of gonadectomy, gonadectomy at a young age has been reported to further increase the risk of CrCLD (Ekenstedt et al., 2017; Hart et al., 2014, 2016; Kieves et al., 2024; Simpson et al., 2019; Torres de la Riva et al., 2013). However, these approaches to categorically classify dogs by gonadectomy status or timing of gonadectomy may be methodologically limited and may potentially bias results. It has been previously suggested that dichotomous binning at the time of data collection risks misclassification and ignores the cumulative lifetime gonadal hormone exposure of an individual dog (Waters et al., 2011). Furthermore, there is no consensus on the definition of early gonadectomy in male or female dogs, whether in general or specifically in the context of CrCLD risk, with varying definitions among studies (Ekenstedt et al., 2017; Hart et al., 2020a; Simpson et al., 2019; Torres de la Riva et al., 2013). Earlier studies have traditionally defined early gonadectomy as that being performed before 6 or 12 months of age. More recent studies have broadened this definition, with one study reporting an increased risk of CrCLD associated with gonadectomy before musculoskeletal maturity at 24 months of age (Waters et al., 2023), and two studies reporting increased longevity in dogs with at least 4.5 years of gonad exposure, compared to dogs gonadectomised before 4.5 years (Joonè & Konovalov, 2023; Waters et al., 2009).

Other studies have used an alternative approach, treating gonadal hormone exposure as a continuous variable. In women, shorter lifetime endogenous gonadal hormone exposure has been associated with areas of increased signal intensity on brain magnetic resonance imaging (Cote et al., 2023), which is a risk factor for cerebrovascular and neurodegenerative disease (Debette & Markus, 2010). In dogs, a lower percentage lifetime exposure to gonadal hormones has been associated with increased fear‐aggressive behaviour (Starling et al., 2019).

Methodologically, categorisation of continuous data may lead to loss of information and thereby mask potential trends within the data (Barrio et al., 2013). In some instances, these cut‐offs are arbitrary, and there may not be a strong physiologic basis to justify these various definitions of early gonadectomy. A previous study investigating gonadal hormone exposure categorically defined a cut‐off for early endocrine disruption as 24 months of age, on the basis of breed‐specific musculoskeletal development (Waters et al., 2023).

Instead of treating continuous data as categorical data, continuous data should be preserved to prevent information loss (Barrio et al., 2013; Bennette & Vickers, 2012). A generalised additive model (GAM) is a regression model, which may be used to capture nonlinear relationships between multiple independent variables and a dependent variable through the use of additive nonlinear smooth terms. A GAM also has the advantage of being easily interpretable in comparison to other non‐parametric models. Data trends in biomedical research are not always linear (Mundo et al., 2022), and GAMs may be used to extend previous research via alternative nonlinear analysis of data (Sørensen et al., 2021). This study aimed to investigate the association between cumulative gonadal hormone exposure as a continuous variable with risk of CrCLD in dogs. Specifically, the following null hypotheses were tested, in that firstly, there is no association between these variables and that secondly, the relationship between cumulative gonadal hormone exposure and CrCLD risk is linear.

## MATERIALS AND METHODS

### Study design and population

Secondary analysis of data from a series of previous studies that investigated the association between age at gonadectomy and various health outcomes in dogs (Hart et al., 2020a, 2020b, 2024) was conducted, with a specific focus on the risk of CrCLD as the outcome measure. The study protocol was preregistered on the Open Science Framework on 16 June 2024 (Low, 2024b), and additional analyses were performed after peer review.

The dataset of this study was obtained via pooling of datasets from the three studies (Hart et al., 2020a, 2020b, 2024), freely available as supplementary files. Full details regarding study population and methodology have been previously published. Briefly, data from a retrospective cohort spanning 36 years (1988 to 2023 inclusive) was obtained from the records of the Veterinary Medical Teaching Hospital (VMTH) at the University of California‐Davis, containing the following information: patient identification number, breed, date of birth, sex, categorical gonadectomy status, date of gonadectomy, date of first hospital visit, date of last hospital visit and presence of any of the 10 diseases of interest. Apart from CrCLD, incidence data on hip dysplasia, elbow dysplasia, lymphoma, mast cell tumour, osteosarcoma, haemangiosarcoma, pyometra, urinary incontinence and mammary cancer were collected. For CrCLD specifically, diagnosis was made at the VMTH through orthopaedic examination, radiography and surgery. There were a total of 45 defined breeds, including five weight categories of mixed‐breed dogs. Invalid disease data, as defined by the original authors, meant that either the disease diagnosis occurred before the study range or that the disease was not confirmed through the appropriate diagnostic criteria. For this study, cases with invalid disease data were classified as non‐diseased.

### Data preprocessing

Date of birth, date of gonadectomy, date of first hospital visit and date of last hospital visit were extracted. Cumulative hormone exposure, in days, was calculated as the difference between date of birth and date of gonadectomy, or the difference between date of birth and date of last hospital visit, for gonadectomised and sexually intact dogs, respectively. The diagnosis of CrCLD disease was extracted as a binary variable. Age at diagnosis of CrCLD, in days, was calculated as the difference between date of birth and date of diagnosis of CrCLD. Age at last follow‐up, in days, was calculated as the difference between date of birth and date of last hospital visit. Incidence data and date of diagnosis of the other nine diseases of interest were collected for covariate analysis. Pyometra, urinary incontinence and mammary cancer incidence data were not included in the statistical analysis for male dogs, as these diseases were either irrelevant or extremely rare. Breed and sex were extracted from raw data without preprocessing.

### Statistical analysis

Datasets for male and female dogs were analysed independently. The diagnosis of CrCLD was binarily defined as the dependent outcome variable. Cumulative hormone exposure was defined as the independent variable of interest. Initially, gonadectomised and intact dogs were compared dichotomously without consideration of cumulative hormone exposure, with comparative CrCLD risk expressed as odds ratios (ORs) and their corresponding 95% confidence intervals (CIs).

During the GAM building process, disease variables other than CrCLD were included as covariates. Multicollinearity was assessed through inspection of the collinearity matrix and calculation of variance inflation factor (VIF) values, with pairwise Pearson correlation coefficient (PCC) greater than 0.5 or VIF values greater than 5 indicative of potential multicollinearity. Collinear variables were removed if either of these conditions was fulfilled. Concurvity values were inspected, and variables with values greater than 0.6 were considered evidence of concurvity; these variables were excluded. A forward stepwise model selection procedure was employed, guided by the Akaike information criterion (AIC) value. Variables were incrementally added to the model based on their contribution to improving model fit while penalising model complexity.

A GAM was fitted for male and female dogs separately, with the outcome modelled as a binary response using a binomial distribution, using a restricted maximum likelihood estimator. Cumulative gonadal hormone exposure was modelled as a smooth term using a cubic spline basis with 12 degrees of freedom. The included covariates were modelled as smooth terms using penalised splines with 6 degrees of freedom. Breed was incorporated as a categorical factor. Estimates of factor term significance were expressed as log‐odds and standard errors (SEs). The final models were assessed by the significance of smooth terms and factor levels. To assess model performance and generalisability, stratified *K*‐fold cross‐validation (*K* = 10) was conducted separately for males and females. The area under the receiver operating characteristic (AUC) curve was calculated for each fold and averaged to obtain a summary measure of model performance. Partial dependence plots were generated to visualise the relationship between cumulative gonadal hormone exposure and the predicted probability of CrCLD, marginalising over the covariates in the model. The predicted probability of CrCLD (*ŷ*) was calculated across a range of hormone exposure values and curve minima were determined by examining the magnitude of *ŷ*. Residual normality was assessed using quantile–quantile (Q–Q) plots. Assuming normality, 95% CIs were calculated with standard errors of the fitted values. Basis dimension adequacy was assessed with the *k*‐index and associated P‐values, with *k*‐indices less than 0.9 considered indicative of inappropriate basis dimensions. Data normality was assessed with the Shapiro–Wilk test. Parametric data were expressed as mean (95% CI) and non‐parametric data were expressed as median (interquartile range). Statistical significance was defined as P‐values of less than 0.05.

Data preprocessing, statistical analyses and visualisation were performed using R version 4.4.1 and Python version 3.10.12. Key R packages included *mgcv* 1.9.1 (Wood, 2017), *caret* 6.0.94 (Kuhn, 2008), *dplyr* 1.1.4 (Wickham et al., 2023), *readr* 2.1.5 (Wickham, Hester, & Bryan, 2024), *tidyr* 1.3.1 (Wickham, Vaughan, & Girlich, 2024), *broom* 1.0.6 (Robinson, 2024), *pdp* 0.8.1 (Greenwell, 2017) and *ggplot2* 3.5.1 (Wickham, 2016); key Python packages included *numpy* 1.26.4 (Harris et al., 2020), *pandas* 2.1.4 (The Pandas Development Team, 2020), *matplotlib* 3.7.1 (Hunter, 2007), *statsmodels* 0.14.2 (Seabold & Perktold, 2010) and *seaborn* 0.13.1 (Waskom, 2021).

## RESULTS

### Study population

The study population consisted of 9845 female dogs (6106 gonadectomised) and 10,745 male dogs (5133 gonadectomised). Thirty dogs were excluded from the original dataset due to invalid demographic data. The median age at gonadectomy was 366 days (184 to 1126 days) and 366 days (184.5 to 1020 days) for females and males, respectively. The most common breeds were the Labrador retriever (*n* = 1933), German shepherd dog (*n* = 1257), golden retriever (*n* = 1247) and Chihuahua (*n* = 1037). Breed distribution, total number of CrCLD cases and incidence rates by breed are presented in File S1, with prevalence rates calculated as the proportion of dogs with CrCLD among the total number of dogs for each breed.

The incidence of CrCLD was 245 of 9845 (2.49%) in females and 203 of 10,745 (1.89%) in males. There were 306 dogs (1.49%) with invalid CrCLD data. The median age at diagnosis of CrCLD was 1945 days (1215 to 2709.5 days) for females and 1581 days (1017 to 2633 days) for males. In 141/448 (31.5%) cases, gonadectomy was not an antecedent event, with a median difference of 185 days (57 to 599 days). For female dogs, the median age at the last hospital visit was 1985 days (1224.5 to 2768 days) and 1703 days (853 to 2851 days) for cases and controls, respectively. For male dogs, the median age at the last hospital visit was 1707 days (1069 to 2684 days) and 1902 days (939 to 3056 days) for cases and controls, respectively. When analysed by dichotomous gonadectomy status, both gonadectomised females (OR = 3.027, 95% CI = 2.166 to 4.231) and gonadectomised males (OR = 1.929, 95% CI = 1.446 to 2.574) had a significantly increased risk of CrCLD, compared to their sexually intact counterparts.

### Statistical modelling

Collinearity diagnostics were performed, and age at last hospital visit was excluded from further analysis with PCCs of 0.51 and 0.66 (Files S2 and S3), and VIFs of 5.31 and 7.48 for females and males, respectively (File S4). Concurvity diagnostics showed no significant correlations between variables (concurvity values all < 0.07). Model selection was performed using a forward stepwise approach based on the Akaike information criterion (AIC). The final model for both females and males included breed, hip dysplasia and elbow dysplasia as covariates.

**FIG 1:**
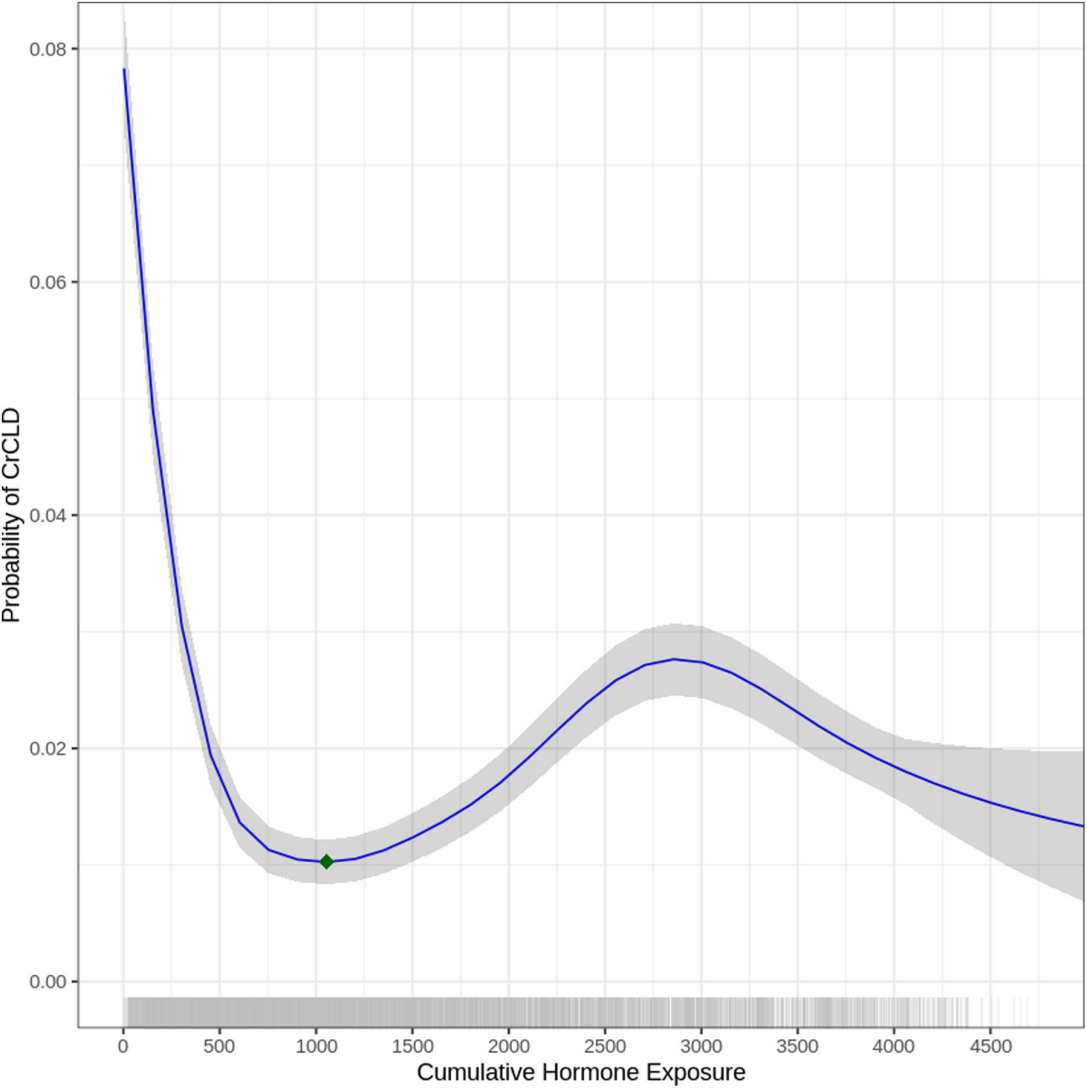
Partial dependence plot and rug plot illustrating the relationship between cumulative gonadal hormone exposure (days) and the predicted probability of cranial cruciate ligament disease for female dogs. Solid lines represent the fitted generalised additive model. Shaded areas represent the 95% confidence interval. Green diamond represents the curve minimum.

**FIG 2:**
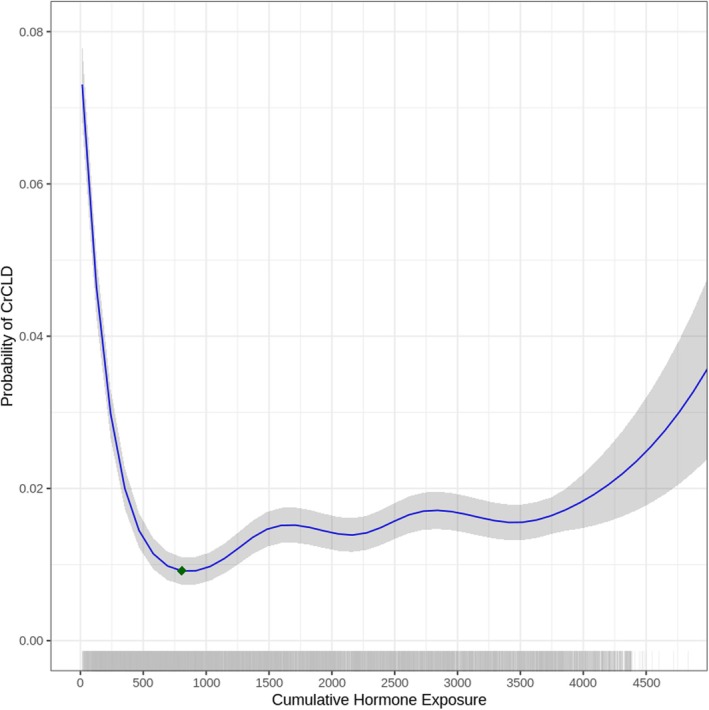
Partial dependence plot and rug plot illustrating the relationship between cumulative gonadal hormone exposure (days) and the predicted probability of cranial cruciate ligament disease for male dogs. Solid lines represent the fitted generalised additive model. Shaded areas represent 95% confidence interval. Green diamond represents the curve minimum.

A GAM was fitted for females and males with cumulative hormone exposure as the independent variable and CrCLD as the outcome of interest, adjusted for the included covariates. Cumulative hormone exposure was significantly associated with the risk of CrCLD, in both females (P < 0.001) and males (P < 0.001). In the final model, the covariates of hip dysplasia (P < 0.001), elbow dysplasia (P < 0.001), the Mastiff breed (estimate = 1.902, SE = 2.879, P = 0.015), the Newfoundland breed (estimate = 2.129, SE = 2.947, P = 0.008) and the Rottweiler breed (estimate = 1.784, SE = 2.751, P = 0.017) were significantly associated with the risk of CrCLD in females. In the final model, the covariates of hip dysplasia (P < 0.001), elbow dysplasia (P < 0.001) and the Newfoundland breed (estimate = 1.566, SE = 2.456, P = 0.019) were significantly associated with the risk of CrCLD in males. The predictive performance of the models was assessed using stratified 10‐fold cross‐validation, with a mean AUC of 0.7706 for females and 0.7617 for males, indicating moderate discriminative ability. The association between cumulative gonadal hormone exposure and the probability of CrCLD was visualised with partial dependence plots in female (Fig 1) and male (Fig 2) dogs. In both sexes, the risk of CrCLD was nonlinearly associated with cumulative gonadal hormone exposure. The risk of CrCLD was greatest in dogs with the least gonadal hormone exposure, sharply decreasing to minima at 1054 days for females and 805 days for males. Residual diagnostics showed no significant deviation from normality. Basis dimensions were assessed to be adequate (*k*‐indices all > 0.9, P‐values all > 0.05).

## DISCUSSION

This study extends the findings of earlier research (Hart et al., 2020a, 2020b, 2024) through alternative statistical modelling of the same dataset, investigating the association between cumulative gonadal hormone exposure as a continuous variable and the risk of CrCLD. Both null hypotheses were rejected in that nonlinear associations were found in both sexes between the duration of gonadal hormone exposure and the risk of CrCLD.

Reanalysis of biomedical data increases the value of research with which to improve clinical practice (Naudet et al., 2018), and is not without precedent in veterinary medicine (Barrett‐Jolley & German, 2024). The findings herein align with other studies in that gonadectomy, and age at gonadectomy, have both been previously shown to be associated with the risk of CrCLD (Adams et al., 2011; Low, 2024a; Pegram et al., 2023; Waters et al., 2023), with dogs gonadectomised at younger ages being at increased risk of CrCLD (Ekenstedt et al., 2017; Simpson et al., 2019; Waters et al., 2023). The mechanisms underpinning this association are likely complex and multifactorial. Gonadectomy leads to persistently elevated luteinising hormone levels through loss of negative feedback mechanisms and this may have direct effects on non‐reproductive luteinising hormone‐bearing tissues, such as the cranial cruciate ligament (Kiefel & Kutzler, 2020; Kutzler, 2023). Physeal closure is mediated by gonadal hormones (Nilsson et al., 2014) and indirectly, gonadectomy may result in dysregulation of physeal growth and an increased tibial plateau angle, which is a known risk factor for CrCLD (Aertsens et al., 2015; Fox et al., 2020). Gonadectomy also decreases resting energy requirements and increases the risk of obesity, which is another known risk factor for CrCLD (Lefebvre et al., 2013; Benka et al., 2023). Socio‐economically, gonadectomised dogs have a longer lifespan (Hoffman et al., 2013) and thus lifetime risk of CrCLD, and are also more likely to have pet insurance (Pegram et al., 2023), which increases access to veterinary care and likelihood of being diagnosed with CrCLD.

Previous studies have investigated age at gonadectomy as a categorical variable, which may be problematic for the following reasons. Firstly, this approach assumes homogeneity of risk within a categorical group (Bennette & Vickers, 2012), which may not be true, especially for defined categories with large age intervals. For example, by defining early gonadectomy as that performed at less than 12 months of age, it is assumed that all dogs in the comparator group (those gonadectomised at more than 12 months of age or sexually intact dogs) have the same risk of CrCLD. Secondly, from an evidence‐based medicine perspective, varying definitions between studies makes direct comparison of reported results challenging. Thirdly, previous statistical approaches assume linearity within the data. For example, the methodology in (Hart et al., 2020a, 2020b, 2024) may have masked nonlinear associations between CrCLD risk and cumulative hormone exposure within certain breeds. Examining raw data from the Bulldog breed as an example, male Bulldogs gonadectomised between 12 and 24 months and female Bulldogs gonadectomised between 6 and 12 months had the lowest incidence of CrCLD, compared to all other groups; however, this was not reflected in the results due to the study’s methodology. Lastly, categorisation, and especially multi‐group categorisation, necessitates repeated multiple pairwise comparisons of groups and increases the risk of Type 1 statistical errors (Ranganathan et al., 2016).

Taking an alternative statistical approach, this study demonstrates that the risk of CrCLD beyond 24 months of age, as a previously defined liberal cut‐off, is neither homogeneous nor linear. In this study, analysis via dichotomous gonadectomy status showed that gonadectomy is associated with an increased risk of CrCLD. This is consistent with previous research but masks the more complex association revealed by the GAM. Furthermore, a high‐risk group can be defined from the data, in that removal of gonadal hormones prior to 1054 days for females and 805 days for males is associated with a substantially increased risk of CrCLD, as seen from the steep curves on the left. Beyond these curve minima, it is noteworthy that the nonlinear risk of CrCLD differs between female and male dogs. This may be due to physiologic differences between females and males, or this may reflect limitations in the data and statistical methods.

The decreasing number of gonadectomy procedures observed at older ages, as evidenced by the left‐skewed distribution of the independent variable, may have influenced the reliability of the model predictions for middle‐ to older‐aged dogs. The non‐randomised nature of elective gonadectomy in this client‐owned population of dogs would also have introduced confounding. For the subset of dogs aged 4 to 8 years of age undergoing elective gonadectomy, some of these dogs may have had this procedure concurrently with surgical treatment of CrCLD, for reasons of convenience. This was inferred from examination of the raw data where dates of gonadectomy were clustered around the date of diagnosis of CrCLD, but this cannot be proven in the absence of clinical records. Dogs undergoing elective gonadectomy also represent a distinct population subset with specific characteristics, such as higher socio‐economic status and potentially different access to veterinary care. In 31% of cases with CrCLD, gonadectomy did not precede the diagnosis of CrCLD, which may dilute the observed association. Therefore, a direct causal link between early gonadectomy and an increased risk of CrCLD cannot be established from this data. These confounding factors may have influenced the observed association between gonadectomy and CrCLD risk and the associations observed in this study.

The incidence of CrCLD, overrepresentation of female dogs, overrepresentation of middle‐aged dogs and breed predispositions align with previous reports (Pegram et al., 2023; Witsberger et al., 2008), and shows that this dataset may be generalised to the general population of dogs. Furthermore, the VMTH is both a first‐opinion and referral hospital, further reinforcing this dataset’s generalisability. The median age at last hospital visit was approximately 4.5 to 5.5 years for both sexes, and this was recognised as a limitation in the original series of studies. Given the retrospective nature of the dataset with non‐standardised follow‐up, all control dogs remain at risk of CrCLD throughout the remainder of their lifetime. Similarly, the reason for gonadectomy, elective or otherwise, was not available from retrospective records. The incidence of CrCLD is relatively low, leading to an imbalance between the case and control groups. This may have affected model performance; although moderate model discriminative ability was demonstrated through the cross‐validation process. Invalid disease data were noted in 306 dogs because of either nonsensical dates of diagnosis of CrCLD or lack of satisfactory diagnosis of CrCLD. These dogs were considered non‐diseased for this study; however, the exclusion of these dogs from the case group may have influenced the results. The age at gonadectomy was also not always based on primarily collected data and may have been based on client reporting, which has been shown to be imperfectly accurate and may have been a source of recall bias (Althubaiti, 2016). Dogs from the original series of studies were also included only if age at gonadectomy was recorded, which may bias the sample population towards dogs from certain geographic areas and socio‐economic groups with better record‐keeping. The original series of studies also did not differentiate between unilateral, metachronous and synchronous orthopaedic disease. In the context of CrCLD, the case group herein thus represents a heterogeneous population of dogs with at least three disease phenotypes. This study also did not perform breed‐specific analyses for CrCLD, unlike the original series of studies. The nature of generalised additive modelling necessitates large datasets to accurately model smooth terms, and it would not have been possible to analyse individual breeds without the risk of overfitting. Despite a large dataset, only moderate model discriminative ability was achieved. Nonetheless, to account for potential breed‐related differences, breed was included as a categorical variable in the analysis and final multivariable models. The secondary analysis was also limited by the primary data in that other clinical variables were unavailable and thus not included in the final model. The original series of studies noted at least 13 breeds where CrCLD risk was not sensitive to gonadectomy. Therefore, the results herein may be more useful at a population level and may have limited direct clinical applicability to dog breeds not at high risk of CrCLD.

This study suggests that gonadectomy is very likely to be associated with an increased risk of CrCLD. The results herein were similar to a recent report where endocrine disruption before 24 months of age was associated with an increased risk of CrCLD (Waters et al., 2023). Despite differences in sample populations and study methodologies, this further reinforces that removal of gonad hormones should be performed after musculoskeletal maturity, if at all. In defense of the categorical approach, categorisation can be useful when translating research findings into clinical practice (Turner et al., 2010), and could be considered a complementary approach. In the context of CrCLD risk, this study may lay the groundwork for a truly empirical definition of early gonadectomy, as inferred from the partial dependence plot curve minima at 1054 and 805 days, for females and males, respectively. It must be emphasised that these findings are preliminary and remain to be externally validated before clinical practice recommendations can be made. Further research should thus replicate these novel findings, and may also seek to empirically define early gonadectomy through a penalised splines GAM approach (Barrio et al., 2013).

In conclusion, this study demonstrates a nonlinear association between cumulative gonadal hormone exposure and the risk of CrCLD in both female and male dogs. The greatest risk of CrCLD was noted before 1054 and 805 days for female and male dogs, respectively. These findings may serve as a preliminary empirical definition of early gonadectomy in dogs in the context of CrCLD risk.

## Author contributions

**D. Low:** Conceptualization (lead); data curation (lead); formal analysis (lead); funding acquisition (lead); investigation (lead); methodology (lead); project administration (lead); resources (lead); software (lead); supervision (lead); validation (lead); visualization (lead); writing – original draft (lead); writing – review and editing (lead).

## Conflict of interest

No conflicts of interest have been declared.

## Funding information

This work has not been presented at any scientific meeting. Support for open access publication fees were funded by IVC Evidensia.

## Supporting information


**File S1.** Breed distribution and cranial cruciate ligament disease incidence rates.

**File S2.** Correlation coefficient matrix of modelled variables for female dogs.

**File S3.** Correlation coefficient matrix of modelled variables for male dogs.

**File S4.** Variance inflation factor of modelled variables for male and female dogs.

**File S5.** Preprocessed raw dataset.

**File S6.** Analytic code in R.

## Data Availability

The raw datasets from the original studies are available at https://figshare.com/s/eaefceb41c81372cafbf. The preprocessed dataset generated from this study is available at File S5. All analytic code used in this study is available at File S6.
