# Supplementary figures and images for: Cumulative gonadal hormone exposure is nonlinearly associated with risk of canine cranial cruciate ligament disease: a generalised additive model analysis of 20,590 dogs (1988‐2023)
Source: J Small Anim Pract. 2025 Aug 21;67(2):122–9. doi: 10.1111/jsap.70023 (PMC12883308; doi:10.1111/jsap.70023)

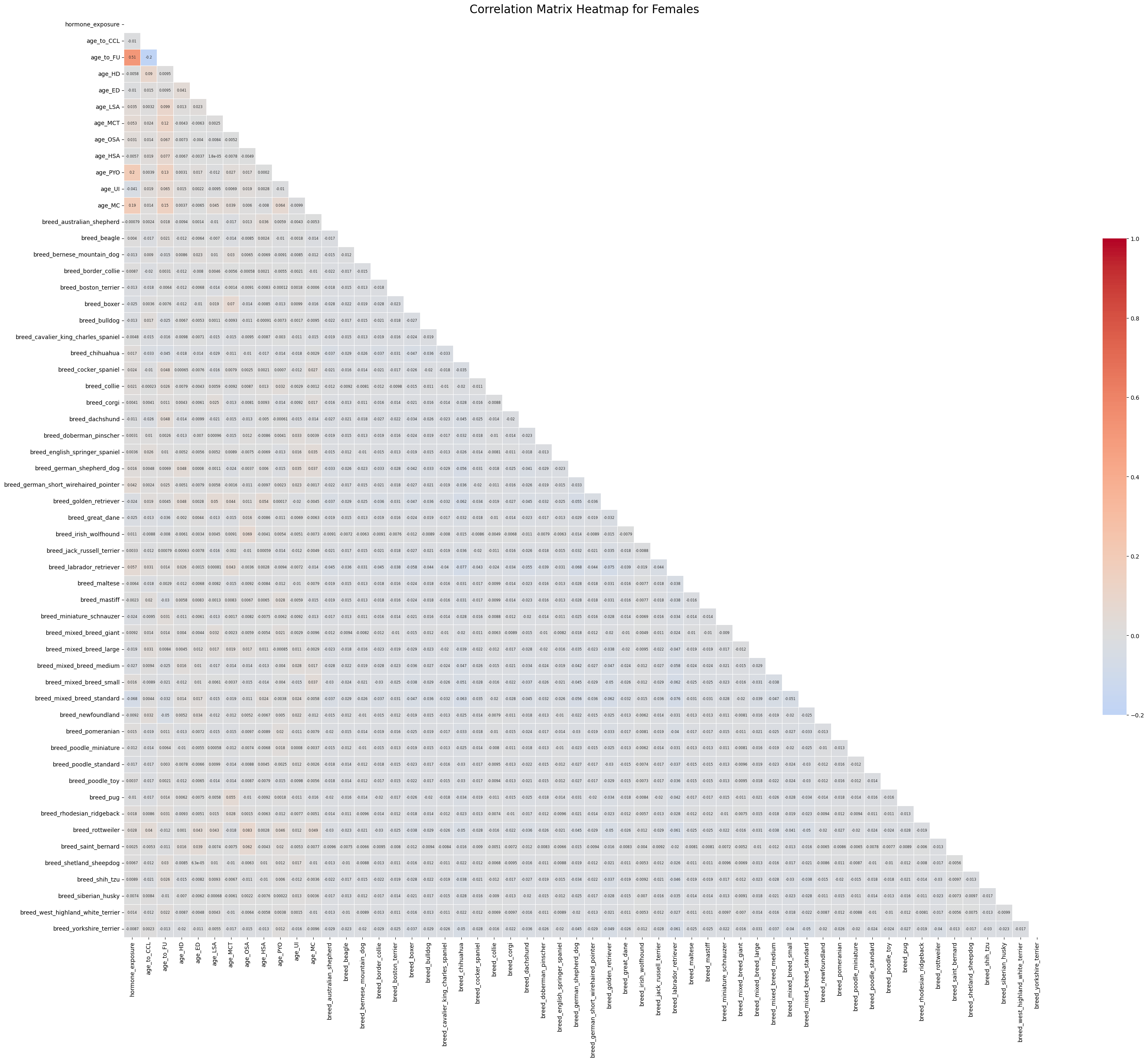

Supplement: Supplementary file 2 — File S2. Correlation coefficient matrix of modelled variables for female dogs. [file JSAP-67-122-s002.png]

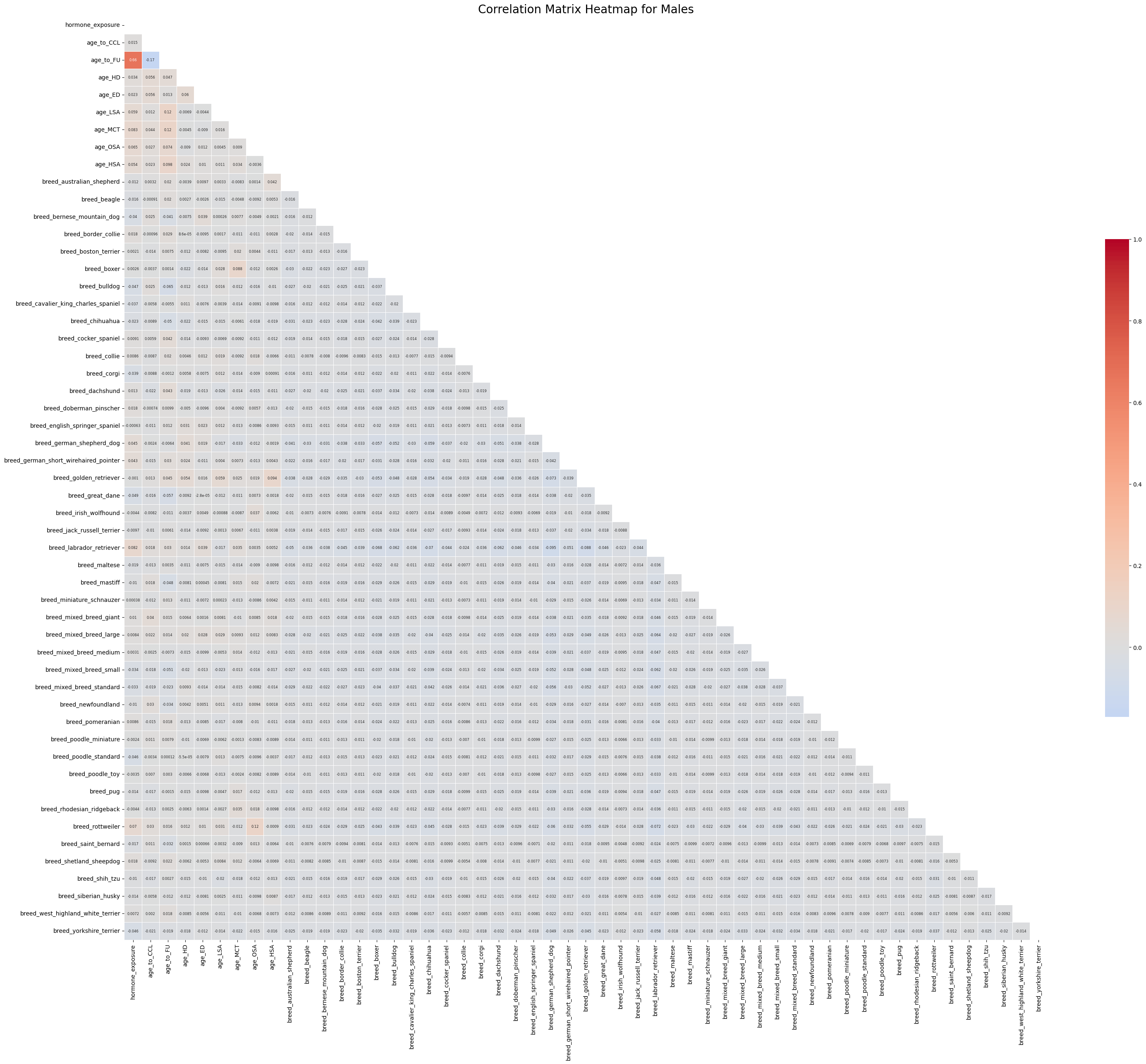

Supplement: Supplementary file 3 — File S3. Correlation coefficient matrix of modelled variables for male dogs. [file JSAP-67-122-s004.png]
