# Supplementary material for: Cumulative gonadal hormone exposure is nonlinearly associated with risk of canine cranial cruciate ligament disease: a generalised additive model analysis of 20,590 dogs (1988‐2023)
Source: J Small Anim Pract. 2025 Aug 21;67(2):122–9. doi: 10.1111/jsap.70023 (PMC12883308; doi:10.1111/jsap.70023)
Supplement: Supplementary file 4 — File S4. Variance inflation factor of modelled variables for male and female dogs. [file JSAP-67-122-s005.pdf]

VIF for Males:

	feature	VIF
0	hormone_exposure	4.577981
1	age_to_CCL	1.102359
2	age_to_FU	7.484465
3	age_HD	1.032519
4	age_ED	1.019083
5	age_LSA	1.047330
6	age_MCT	1.052681
7	age_OSA	1.035071
8	age_HSA	1.033477
9	breed_australian_shepherd	1.075519
10	breed_beagle	1.043970
11	breed_bernese_mountain_dog	1.026940
12	breed_border_collie	1.067086
13	breed_boston_terrier	1.041364
14	breed_boxer	1.121650
15	breed_bulldog	1.065796
16	breed_cavalier_king_charles_spaniel	1.033957
17	breed_chihuahua	1.091866
18	breed_cocker_spaniel	1.074377
19	breed_collie	1.020915
20	breed_corgi	1.034606
21	breed_dachshund	1.124468
22	breed_doberman_pinscher	1.059684
23	breed_english_springer_spaniel	1.035740
24	breed_german_shepherd_dog	1.220641
25	breed_german_short_wirehaired_pointer	1.082427
26	breed_golden_retriever	1.238526
27	breed_great_dane	1.028697
28	breed_irish_wolfhound	1.012077
29	breed_jack_russell_terrier	1.051183
30	breed_labrador_retriever	1.346481
31	breed_maltese	1.035454
32	breed_mastiff	1.040027
33	breed_miniature_schnauzer	1.034757
34	breed_mixed_breed_giant	1.067376
35	breed_mixed_breed_large	1.115736
36	breed_mixed_breed_medium	1.054319
37	breed_mixed_breed_small	1.067479
38	breed_mixed_breed_standard	1.097248
39	breed_newfoundland	1.024074
40	breed_pomeranian	1.049859
41	breed_poodle_miniature	1.032220
42	breed_poodle_standard	1.040530
43	breed_poodle_toy	1.029901
44	breed_pug	1.053905
45	breed_rhodesian_ridgeback	1.034463
46	breed_rottweiler	1.164348
47	breed_saint_bernard	1.008806
48	breed_shetland_sheepdog	1.022742

49	breed_shih_tzu	1.061275
50	breed_siberian_husky	1.034029
51	breed_west_highland_white_terrier	1.025215
52	breed_yorkshire_terrier	1.076058

VIF for Females:

	feature	VIF
0	hormone_exposure	3.160349
1	age_to_CCL	1.105553
2	age_to_FU	5.307209
3	age_HD	1.030436
4	age_ED	1.013526
5	age_LSA	1.038658
6	age_MCT	1.053239
7	age_OSA	1.032666
8	age_HSA	1.022997
9	age_PYO	1.068362
10	age_UI	1.030772
11	age_MC	1.071045
12	breed_australian_shepherd	1.070306
13	breed_beagle	1.046116
14	breed_bernese_mountain_dog	1.026846
15	breed_border_collie	1.060043
16	breed_boston_terrier	1.038123
17	breed_boxer	1.094922
18	breed_bulldog	1.049617
19	breed_cavalier_king_charles_spaniel	1.040503
20	breed_chihuahua	1.136721
21	breed_cocker_spaniel	1.075997
22	breed_collie	1.025736
23	breed_corgi	1.039919
24	breed_dachshund	1.116491
25	breed_doberman_pinscher	1.049423
26	breed_english_springer_spaniel	1.036408
27	breed_german_shepherd_dog	1.145996
28	breed_german_short_wirehaired_pointer	1.075493
29	breed_golden_retriever	1.170595
30	breed_great_dane	1.031054
31	breed_irish_wolfhound	1.014223
32	breed_jack_russell_terrier	1.056835
33	breed_labrador_retriever	1.274233
34	breed_maltese	1.041424
35	breed_mastiff	1.035614
36	breed_miniature_schnauzer	1.047537
37	breed_mixed_breed_giant	1.024454
38	breed_mixed_breed_large	1.073894
39	breed_mixed_breed_medium	1.083564
40	breed_mixed_breed_small	1.098838
41	breed_mixed_breed_standard	1.138673
42	breed_newfoundland	1.020840
43	breed_pomeranian	1.053326
44	breed_poodle_miniature	1.029017

45	breed_poodle_standard	1.038991
46	breed_poodle_toy	1.040144
47	breed_pug	1.058412
48	breed_rhodesian_ridgeback	1.037028
49	breed_rottweiler	1.121695
50	breed_saint_bernard	1.015844
51	breed_shetland_sheepdog	1.029657
52	breed_shih_tzu	1.072910
53	breed_siberian_husky	1.032557
54	breed_west_highland_white_terrier	1.028226
55	breed_yorkshire_terrier	1.099533
