# Supplementary material for: Cumulative gonadal hormone exposure is nonlinearly associated with risk of canine cranial cruciate ligament disease: a generalised additive model analysis of 20,590 dogs (1988‐2023)
Source: J Small Anim Pract. 2025 Aug 21;67(2):122–9. doi: 10.1111/jsap.70023 (PMC12883308; doi:10.1111/jsap.70023)
Supplement: Supplementary file 6 — File S6. Analytic code in R. [file JSAP-67-122-s006.pdf]

```r
install.packages("pdp")
install.packages("ggplot2")
install.packages("mgcv")
install.packages("caret")
install.packages("pROC")
install.packages("glmnet")
```

```r
library(pdp)
library(ggplot2)
library(mgcv)
library(splines)
library(caret)
library(pROC)
library(glmnet)
library(dplyr)
library(tidyr)
library(readr)
library(broom)
```

```r
data_M <- read_csv(temp_file_M)
data_F <- read_csv(temp_file_F)
```

```r
# Incidence calculation
data_M$CCL_disease <- as.numeric(as.character(data_M$CCL_disease))
data_F$CCL_disease <- as.numeric(as.character(data_F$CCL_disease))

incidence_by_breed_M <- data_M %>%
  group_by(breed) %>%
  summarize(
    total_count = n(),
    ccld_count = sum(CCL_disease),
    incidence = ccld_count / total_count
  )

incidence_by_breed_F <- data_F %>%
  group_by(breed) %>%
  summarize(
    total_count = n(),
    ccld_count = sum(CCL_disease),
    incidence = ccld_count / total_count
  )
```

```r
print(incidence_by_breed_M, n = 45)
print(incidence_by_breed_F, n = 45)
```

```r
# Concurvity check
model_concurvity_M <- gam(CCL_disease ~ s(hormone_exposure, bs = "cs",
k = 12) +
                      s(age_to_CCL, bs = "ps", k = 4) +
                      s(age_HD, bs = "ps", k = 4) +
                      s(age_ED, bs = "ps", k = 4) +
                      s(age_LSA, bs = "ps", k = 4) +
                      s(age_MCT, bs = "ps", k = 4) +
                      s(age_OSA, bs = "ps", k = 4) +
                      s(age_HSA, bs = "ps", k = 4) +
                      factor(breed),
                      data = data_M,
                      family = binomial(),
                      method = "REML")

model_concurvity_F <- gam(CCL_disease ~ s(hormone_exposure, bs = "cs",
k = 12) +
                      s(age_to_CCL, bs = "ps", k = 4) +
                      s(age_HD, bs = "ps", k = 4) +
                      s(age_ED, bs = "ps", k = 4) +
                      s(age_LSA, bs = "ps", k = 4) +
                      s(age_MCT, bs = "ps", k = 4) +
                      s(age_OSA, bs = "ps", k = 4) +
                      s(age_HSA, bs = "ps", k = 4) +
                      s(age_PYO, bs = "ps", k = 4) +
                      s(age_UI, bs = "ps", k = 4) +
                      s(age_MC, bs = "ps", k = 4) +
                      factor(breed),
                      data = data_F,
                      family = binomial(),
                      method = "REML")

# Check for concurvity
concurvity_results_M <- concurvity(model_concurvity_M, full = TRUE)
concurvity_results_F <- concurvity(model_concurvity_F, full = TRUE)

# Print the concurvity results
print(concurvity_results_M)
print(concurvity_results_F)
```

```r
# Stepwise selection
model_stepwise_M <- CCL_disease ~ s(hormone_exposure, bs = "cs", k = 
12) +
                    factor(breed)

model_stepwise_F <- CCL_disease ~ s(hormone_exposure, bs = "cs", k = 
12) +
                    factor(breed)

# Fit the initial model
model_M <- gam(model_stepwise_M, data = data_M, family = binomial(), 
method = "REML")
model_F <- gam(model_stepwise_F, data = data_F, family = binomial(), 
method = "REML")

# Print AIC of the initial model
aic_M <- AIC(model_M)
print(paste("AIC for initial model (Males):", aic_M))
aic_F <- AIC(model_F)
print(paste("AIC for initial model (Females):", aic_F))

# Repeat as necessary
```

```r
# Fit the GAMs
model_M <- gam(model_formula_M, data = data_M, family = binomial(), 
method = "REML", select = TRUE)
model_F <- gam(model_formula_F, data = data_F, family = binomial(), 
method = "REML", select = TRUE)
print(summary(model_M))
print(summary(model_F))
```

```r
# Function to perform stratified K-fold cross-validation
n_folds <- 10
perform_cv <- function(data, formula, n_folds) {
  set.seed(123) # For reproducibility

  # Create stratified folds
  folds <- createFolds(data$CCL_disease, k = n_folds, list = TRUE, 
returnTrain = TRUE)

  aucs <- c()

  for (i in 1:n_folds) {
```

```
    train_indices <- folds[[i]]
    test_indices <- setdiff(1:nrow(data), train_indices)

    train_data <- data[train_indices, ]
    test_data <- data[test_indices, ]

    # Fit the model with shrinkage smoothers
    model <- gam(formula, data = train_data, family = binomial(),
method = "REML", select = TRUE)

    # Make predictions on the test data
    preds <- predict(model, newdata = test_data, type = "response")

    # Compute AUC
    roc_obj <- roc(test_data$CCL_disease, preds)
    auc_value <- auc(roc_obj)

    aucs <- c(aucs, auc_value)
  }

  return(aucs)
}
```

```
# Perform cross-validation
auc_males <- perform_cv(data_M, model_formula_M, n_folds)
mean_auc_males <- mean(auc_males)
print(paste("Cross-Validation AUC for Males: ", mean_auc_males))
auc_females <- perform_cv(data_F, model_formula_F, n_folds)
mean_auc_females <- mean(auc_females)
print(paste("Cross-Validation AUC for Females: ", mean_auc_females))
```

```
# PDP
pdp_M <- partial(model_M, pred.var = "hormone_exposure", prob = TRUE,
plot = FALSE, plot.engine = "ggplot2")
pdp_F <- partial(model_F, pred.var = "hormone_exposure", prob = TRUE,
plot = FALSE, plot.engine = "ggplot2")
```

```
# Minima calculation
nadir_M <- pdp_M %>%
  filter(yhat == min(yhat))
nadir_F <- pdp_F %>%
  filter(yhat == min(yhat))
```

```
print(nadir_M)
print(nadir_F)
```

```
# Plot and print PDPs
p1 <- ggplot(pdp_M, aes(x = hormone_exposure, y = yhat)) +
  geom_line(color = "blue") +
  geom_ribbon(aes(ymin = ci_low, ymax = ci_high), alpha = 0.2) +
  geom_rug(data = data_M, aes(x = hormone_exposure), sides = "b", color
= "grey", size = 0.08, alpha = 0.8, inherit.aes = FALSE) +
  geom_point(data = nadir_M, aes(x = hormone_exposure, y = yhat), color
= "darkgreen", size = 3, shape = 18) +
  labs(x = "Cumulative Hormone Exposure",
       y = "Probability of CrCLD") +
  theme_bw() +
  scale_x_continuous(breaks = seq(0, 4500, by = 500)) +
  coord_cartesian(xlim = c(0, 4750), ylim = c(0, 0.08))

p2 <- ggplot(pdp_F, aes(x = hormone_exposure, y = yhat)) +
  geom_line(color = "blue") +
  geom_ribbon(aes(ymin = ci_low, ymax = ci_high), alpha = 0.2) +
  geom_rug(data = data_F, aes(x = hormone_exposure), sides = "b", color
= "grey", size = 0.08, alpha = 0.8, inherit.aes = FALSE) +
  geom_point(data = nadir_F, aes(x = hormone_exposure, y = yhat), color
= "darkgreen", size = 3, shape = 18) +
  labs(x = "Cumulative Hormone Exposure",
       y = "Probability of CrCLD") +
  theme_bw() +
  scale_x_continuous(breaks = seq(0, 4500, by = 500)) +
  coord_cartesian(xlim = c(0, 4750), ylim = c(0, 0.08))

print(p1)
print(p2)

gam.check(model_M)
gam.check(model_F)
```
